# Analysis of the Prognostic Significance and Immune Infiltration of the Amino Acid Metabolism-Related Genes in Colon Adenocarcinoma

**DOI:** 10.3389/fgene.2022.951461

**Published:** 2022-08-10

**Authors:** Zhenling Wang, Changzhi Huang, Jingyu Wu, Hongqiang Zhang, Yu Shao, Zan Fu

**Affiliations:** ^1^ The First College of Clinical Medicine, Nanjing Medical University, Nanjing, China; ^2^ Department of General Surgery, The First Affiliated Hospital of Nanjing Medical University, Nanjing, China

**Keywords:** amino acid metabolism-related genes, colon adenocarcinoma, LASSO analysis, prognosis, immune infiltration

## Abstract

Amino acid metabolization is verified to be a part in the progression of cancer. However, genes related to the amino acid metabolism have not been identified in colon adenocarcinoma (COAD). A systematic prognostic model of COAD becomes a pressing need. Among genes related to the amino acid metabolism, RIMKLB, ASPG, TH, MTAP, AZIN2, PSMB2, HDC, ACMSD, and PSMA8 were identified to construct a risk model. Kaplan–Meier (K–M) analyses demonstrated that the high-risk group achieved a poor prognosis. Area under the respective ROC (AUC) values indicated the robustness of the model. To highlight its clinical value, multivariate Cox was used to obtain the optimal variables to construct a nomogram. A higher tumor mutation burden was observed in the high-risk group. However, the low-risk group had a stronger immune infiltration. Seven molecular subtypes were found by consensus cluster. Twenty-two hub genes were identified related to the ESTIMATE score using WGCNA. In brief, our research constructed a stable prognostic model related to the amino acid metabolism in COAD, revealing its connection to the immune microenvironment. The model guided the outcome of COAD and the direction of immunotherapy.

## Introduction

Colorectal cancer (CRC) is one of the most prevalent carcinomas and is the third cause of cancer-related death globally. Annually, more than 1.85 million occurring cases of colorectal carcinoma increase, among which 850000 end in death (Biller and Schrag, 2021). The incidence of colorectal cancer varies widely between different geographic regions owing to various risk factors, screening modalities, and access to health care (Favoriti et al., 2016). COAD, the main type of colorectal cancer, shows a younger trend recently (Siegel et al., 2020). The prognosis of COAD has been improved significantly as breakthroughs in surgical technique and adjuvant treatment emerged (Jahanafrooz et al., 2020). However, the 5-year overall survival (OS) is still less than 40% in colon cancer with advanced stage, which is to blame for the postoperative recurrence and metastasis (Mejri et al., 2018). Recognized as the prognostic indicator, the American Joint Committee on Cancer (AJCC) TNM staging system is updated continually, while patients with analogous clinicopathologic features share different prognoses (Lino-Silva et al., 2020). Thence, the discovery of new prognostic markers in colon cancer has become a key point.

The recoding of energy metabolism has been considered a hallmark of cancer (Hanahan and Weinberg, 2011). Proliferation, invasion, and metastasis of cancer are tied to a series of biological processes, in which energy metabolic reprogramming plays a critical role (Boroughs and DeBerardinis, 2015). Amino acid and its metabolism not only participate in protein synthesis but are also involved in metabolic reprogramming to regulate the proliferation of cancer by variable pathways (Li and Zhang, 2016). For example, asparagine synthetase (ASNS) is involved in the synthesis of aspartic acid to asparagine. The silence of ASNS has been verified as an origin of tumor-specific auxotrophy (Vettore et al., 2020). In addition, BCAT1 catalyzes the transamination of branched-chain amino acids (BCAAs) to α-ketoglutarate (α-KG), and it is confirmed to have a positive correlation with a high expression of c-Myc, thereby supporting cell invasion (Zhou et al., 2013). Moreover, glutamine synthetase (GS) synthesizes glutamine using glutamate and NH4+, which is of great importance to continued tumor proliferation, especially when glutamine may be limiting (Tardito et al., 2015). All these results verify that amino acid metabolism is of vital importance in the metabolism reprogramming of carcinoma.

Amino acid metabolism closely participates in the development of colon cancer with variable pathways. For instance, this study proves that SNX10 controls mTOR activation in CRC by controlling the amino acid metabolism depending on CMA (Le et al., 2018). Furthermore, it is universally known that aspartate turns into asparagine through ASNS. CRC cells with mutated KRAS are capable of becoming accustomed to glutamine consumption by the overexpression of ASNS (Toda et al., 2016). Similarly, SLC25A22, a gene inducing intracellular synthesis of aspartate, can promote proliferation in KRAS-mutant CRC cells (Wong et al., 2016). As is shown earlier, the glutamine metabolism plays an outstanding role in colorectal cancer. In addition, other amino acid metabolism pathways also play a part that cannot be underestimated. CircMYH9 promotes the growth of CRC by regulating the metabolism of serine/glycine and reactive oxygen species (ROS) in a p53-dependent way (Liu X. et al., 2021). Thus, targets for the metabolism of COAD patients are under exploration. A recent study shows that a combination of blockade of EGFR and glutamine metabolism shows a new direction of therapy for advanced metastatic COAD (Cohen et al., 2020). Recently, plenty of studies have been focused on the immune microenvironment of colon carcinoma (Li et al., 2020; Geng et al., 2021; Yan et al., 2021). In addition to TNM staging, the assessment of colon cancer recurrence and mortality also needs to include the degree of immune cell infiltration (Galon et al., 2006; Mlecnik et al., 2011). The regulation of immune microenvironment to tumor cells can even determine the outcome of tumor (Yuan et al., 2021). However, based on the importance of the amino acid metabolism, a lack of the evaluation model for amino acid metabolism-related gene signatures on the prognosis of colon carcinoma still exists, whose connection with the immune microenvironment of colon carcinoma has not been confirmed.

## Materials and Methods

### Datasets

Gene expression quantification data (FPKM and counts format) for TCGA-COAD and corresponding statistics on survival and clinical outcomes were obtained from the UCSC Xena browser (http://xena.ucsc.edu/), from which 453 cases of COAD tumor tissues and 41 cases of normal tissues were extracted. Among them, HTSeq-FPKM of 430 COAD samples with survival data were converted to log2(FPKM+1) formation for subsequent analysis. HTSeq-counts were utilized for differential expression profiling. GSE17538 datasets along with clinical data obtained from https://www.ncbi.nlm.nih.gov/geo/ were used as the external validation set, which included 232 tumor samples with OS.

A total of 374 genes related to amino acid metabolic processes were obtained from the MSigDB team (REACTOME_METABOLISM_OF_AMINO_ACIDS_AND_DERIVATIVES): http://software.broadinstitute.org/gsea/index.jsp (Subramanian et al., 2005). The entire process was conducted by R (version 4.1.1).

### Gene Ontology Analysis of the Differentially Expressed Genes in the Normal and Tumor Tissue Samples

Gene expression fold-change between tumor and healthy tissues was accessed for each gene using the “Desq2” R package. Adjusted *p*-value < 0.05 and FC > 1.5 were the screening criteria of genes enrolled for further analysis. The “clusterProfiler” R package was carried out to perform GO analyses on DEGs, which is to describe the roles that genes and proteins play inside cells. The *p*-value along with the q-value <0.05 was believed as a statistical significance. The “GOplot” R package was utilized to achieve the results.

### Construction and Verification of a Prognostic Gene Model

The training and test sets were TCGA-COAD and GSE17538, respectively. In the training set, univariate Cox regression analyses were first implemented with a criterion of “*p* < 0.05.” Multivariable Cox regression using stepwise selection modeling was also applied to further select predictive genes. All Cox regression models were established by the “survival” R package. The “glmnet” R package was for LASSO analysis, which ultimately obtained the most useful predictive genes and their coefficients. The risk score of each sample based on genes screened was reckoned utilizing the following formula:

$$\text{RiskScore}=\sum_{n=1}^{i}\left({\text{Coefi}}^{*}\text{ExpGenei}\right)$$

“Coef” was referred to the regression coefficient derived from the LASSO analysis, and “ExpGene” represented the expression of the selected genes. In TCGA-COAD, we obtained the risk score of each sample and divided the cohort into two groups according to the median risk score. Each group contains 215 patients. K–M curves using the log-rank test were applied to compare the survival probability of patients between the two groups. The time-dependent ROC was drawn employing the “timeROC” R package to appraise the accuracy of the prognostic model. In GSE17538, the formula was applied to distinguish between high (*n* = 111) and low (*n* = 121) groups. K–M plots were implemented to further validate previous results. Finally, the applicability of the model was confirmed to be valid in the external validation set.

### Validation of the Prognostic Gene Model in Clinical Subgroups and Univariable and Multivariate Cox Regressions With Clinical Features

Stratified by the clinicopathological index, K–M curves were plotted to explore the feasibility of the model for different subgroups of patients. Patients were divided into two categories based on age >65 years and age ≤65 years, female and male, T1+T2 and T3+T4, N0 and N1+N2, M0 and M1, and Stages I + II and Stages III + IV, respectively. Then the *p*-value was calculated in K–M curves between the two groups in each category so as to confirm the applicability of risk scores. To better perform Cox regression, samples with missing clinical information have been removed. “Tis” and “Mx” were deleted to acquire a clear stratification. A total of 375 samples were included in the follow-up clinical related analysis.

### Development and Evaluation of a Nomogram for Predicting the Overall Survival

A nomogram depending on the result of multivariable regression was drawn. We construct the figure using the “rms” package to calculate the 1-, 3-, and 5-year survival rates of COAD. To assess the discrimination and accuracy of the nomogram, the ROC diagrams and the calibration curves were plotted. The DCA (decision curve analysis) plotted through the “ggDCA” package was performed to assess the suitability of clinical application and help guide clinicians in decision-making. We use the “ggalluvial” R package to draw an alluvial plot to better reveal the flow of each patient with different clinicopathological features.

### Functional and Pathway Enrichment Analysis

DEGs in low- and high-risk groups were achieved using the “DESeq2” R package with the standard of FDR <0.05 and log2 fold change ≥1. The GO analysis and Kyoto Encyclopedia of Genes and Genomes (KEGG) pathway enrichment analysis were conducted with the “clusterProfiler” package. To further analyze gene set differences between the two groups, we conducted gene set enrichment analysis (GSEA) with *p* < 0.05, FDR <0.25, and NES value >1.

### Estimation of the Immune Cell Infiltration

The association between risk grouping and the infiltration of immune cells was in estimation. We performed three methods: ESTIMATE, CIBERSORT, and ssGSEA, utilizing R package “estimate,” “GSVA.” ESTIMATE shows the immune and stromal scores of the specimen through RNA-seq data based on ssGSEA. CIBERSORT is a deconvolution algorithm that uses gene expression profiling data to count the abundance of 22 types of immune cells. In every sample, the total of 22 immune cell type fractions was 100%. Single sample gene set enrichment analysis (ssGSEA) was performed to quantify the tumor-infiltrating immune cell subgroups and immune function between the two groups, among which 28 types of immune cells were quantified.

### TMB Analysis, Protein–Protein Interaction Network, and Consensus Clustering of the Screened Nine Genes

TMB was calculated as mutations per megabase (mut/Mb), which leads a unique role in mediating antitumor immunity. A total of 399 samples with mutation information were classified into two groups in line with the prognostic model. The “maftools” R package was utilized to perform TMB analysis. The PPI network between the nine genes was performed on GeneMANIA (https://genemania.org). Consensus unsupervised clustering was performed according to the expression of the nine genes, and different molecular subtypes were obtained using the R package “ConsensusClusterPlus.”

### Drug Sensitivity Analysis

To explore the clinical value of chemotherapeutic drugs, the “pRRophetic” package was utilized to calculate semi-inhibitory concentration (IC50) values for common drugs.

### Weighted Gene Co-Expression Network Analysis

We conducted WGCNA utilizing the “WGCNA” R package (Langfelder and Horvath, 2008). We selected 2 as the soft power to fit the standard of the scale-free distribution. Five modules were acquired, and their relationship with risk score, stromal score, immune score, ESTIMATE score, and tumor purity was plotted. Twenty-two immune-related hub genes were obtained with appropriate values of module membership (MM) and gene significance (GS).

### Statistical Analysis

The Wilcoxon rank-sum test was performed to calculate the difference between the two groups. The Kruskal–Wallis test was performed to compare three or more groups. The Spearman test was used to identify correlations between genes screened. The Kaplan–Meier method was utilized to evaluate the association between the two groups. Univariate and multivariate Cox regressions distinguished whether the risk score, age, gender, stage, T, N, and M can become independent prognostic factors. All of the statistical analyses were conducted using R 4.1.1 (*p* < 0.05).

## Results

### Flowchart of the Study

**FIGURE 1:**
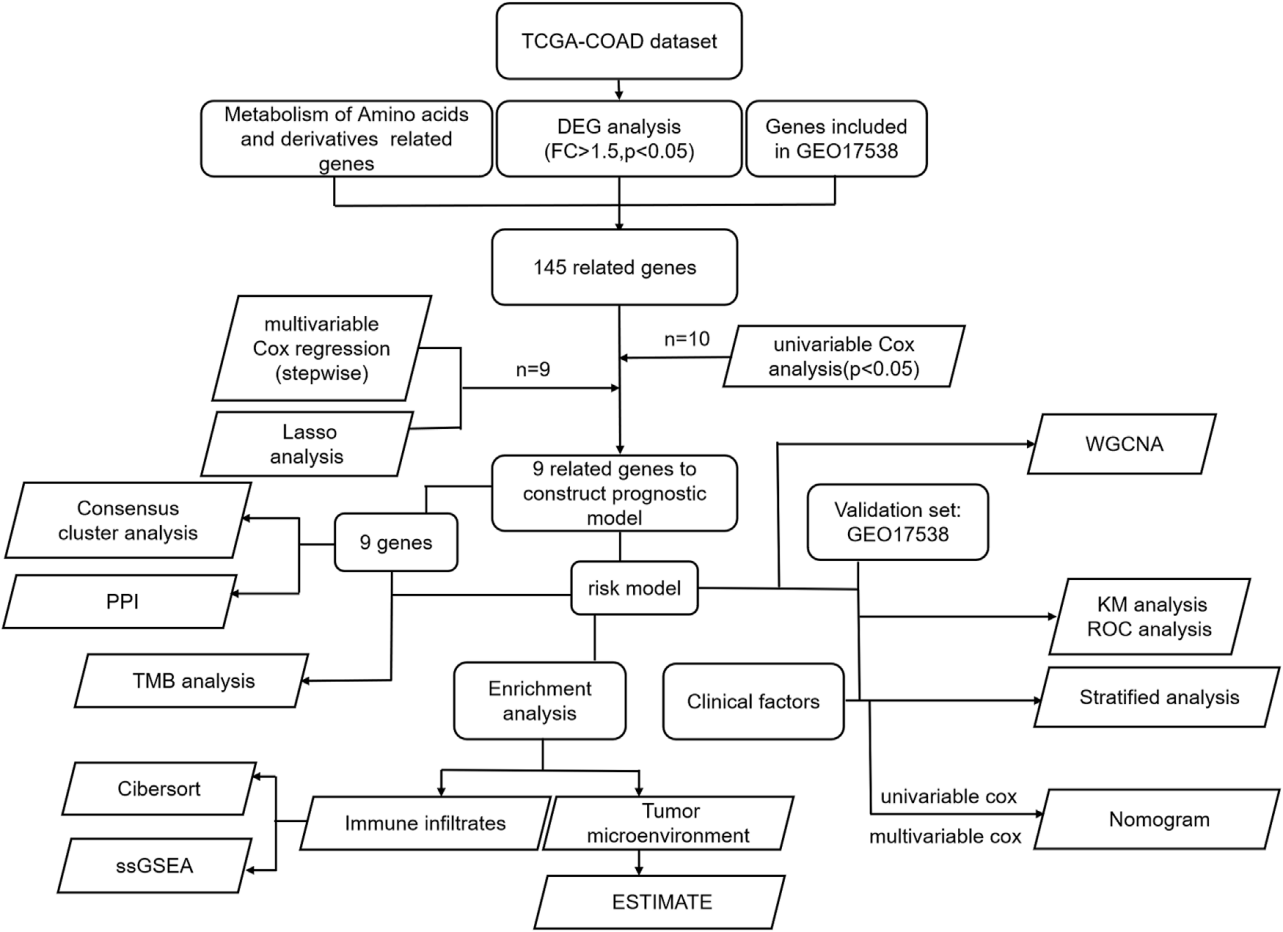
All the procedures of this study.

RNA-seq along with clinically relevant data was obtained from the TCGA-COAD dataset. The common genes in the DEGs of TCGA-COAD and genes in GEO17538 were extracted and intersected with the amino acid metabolism gene set enriched by GSEA to obtain 145 related genes. The genes were screened to nine by univariate and multivariate regression models, and finally, a prognostic model based on nine genes was constructed by LASSO regression. First, we utilized these nine genes to analyze the protein-to-protein interaction network, along with the TMB situation, and intend to explore the prognostic value between groups by consensus clustering. Second, the K–M and ROC curves are drawn according to the risk model. Combined with clinically relevant data, the significance of the K–M curve between each group was discussed in groups, and then a nomogram was constructed. We verified it in the GEO database. Finally, GO analysis, KEGG analysis, and GSEA were performed using the risk model. According to the enriched pathways, we chose to perform subsequent immune-related analysis (Figure 1).

### Identification of Common Genes Related to the Metabolism of Amino Acids in Differentially Expressed Genes of TCGA-COAD

**FIGURE 2:**
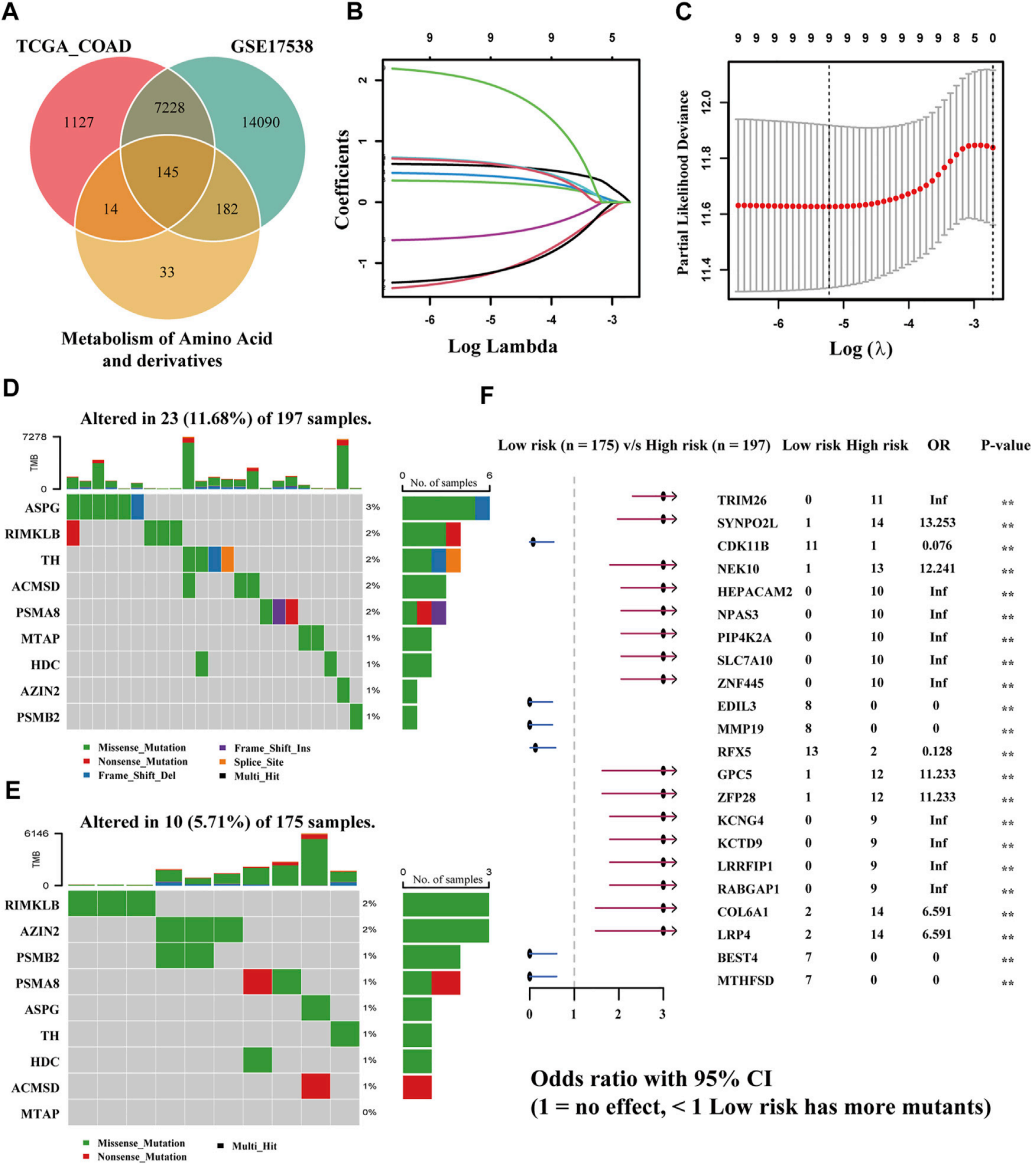
Establishment of a risk model according to amino acid metabolism-related genes in TCGA-COAD and corresponding TMB analysis. **(A)** Intersection of DEGs of TCGA-COAD and GSE17538 and amino acid metabolism-related genes. **(B,C)** LASSO analysis to obtain the nine gene signatures and corresponding coefficients to build the risk model. **(D,E)** TMB of high- and low-risk groups. **(F)** Comparison of the significant mutation genes between the two groups.

First, 453 tumor and 41 normal samples with expression profile (counts format) downloaded from the TCGA-COAD were subjected to differential analysis (FC > 1.5, adjusted *p*-value <0.05), and 8,514 differentially expressed genes were obtained. Then genes were subjected to the GO enrichment analysis, and the following meaningful metabolic pathways were achieved (p-value<0.05, q-value<0.05) (Supplementary Figure S1A; Supplementary Table S1): amine transport, amino acid transport, amino acid transmembrane transport, amino acid import, and amino acid transmembrane transporter activity. It was revealed that the metabolism of amino acids occupied an important part in the progression of colon cancers. The obtained differentially expressed genes were intersected with the amino acid and derivative metabolism-related genes obtained from the GSEA official website and then combined with all the genes in the validation set GEO17538 expression profile. A total of 145 amino acid metabolism-related genes were intersected (Figure 2A). Second, we eliminated 23 tumor samples without survival data, and the remaining 430 samples were used for the subsequent Cox regression analysis. The screening standard of univariate Cox regression is *p* < 0.05, and 10 genes with related HR values ​​are obtained (Supplementary Table S2). Then, multivariate Cox regression utilizing the stepwise method was carried out, and gene DUOX1 was deleted (Supplementary Table S2). The final nine genes (*RIMKLB*, *ASPG*, *TH*, *MTAP*, *AZIN2*, *PSMB2*, *HDC*, *ACMSD*, and *PSMA8*) were obtained by LASSO regression. Among them, *TH*, *MTAP*, *PSMB2*, and *ACMSD* are highly expressed in COAD samples, while *RIMKLB*, *ASPG*, *AZIN2*, *HDC*, and *PSMA8* are on the contrary (Supplementary Figure S1B).

### Establishment of a Prognostic Model in the Training Set and TMB Analysis

The risk model was created by the LASSO regression analysis (Figures 2B,C). The risk score of each sample was obtained utilizing the following formula: (0.601934345258193) * *RIMKLB* + (−1.22749614731757) * *ASPG* + (0.331574905784151) * *TH* + (0.440528442325098) * *MTAP* + (0.665293956039793) * *AZIN2* + (−0.559861290284593) * *PSMB2* + (−1.19852726039675) * *HDC* + (0.650942798766232) * *ACMSD* + (1.99376791470084) * *PSMA8*. The samples in TCGA-COAD were bisected into two equal groups (*n* = 215) based on the median risk score, and the optimal cutoff value is 0.5464642. Subsequently, the somatic mutation profiles of the two groups were drawn separately (Figure 2E). Among 197 samples in the high-risk group, 23 (11.68%) were mutated, while only 10 (5.71%) of 175 in the low-risk group were mutated, which demonstrated that the group scoring more had a higher rate of mutation. Among genes screened, ASPG was the gene with the highest mutation frequency in the group scoring higher; however, it had a less mutation rate in the group scoring lower. RIMKLB has a relatively high mutation frequency in both groups, while MTAP had the lowest mutation frequency. Then, the comparison of differentially mutated genes between the two groups was displayed with a forest plot (*p* < 0.005), and 22 significantly mutated genes are shown in Figure 2F.

### Prognostic Evaluation of the Training and Validation Sets

**FIGURE 3:**
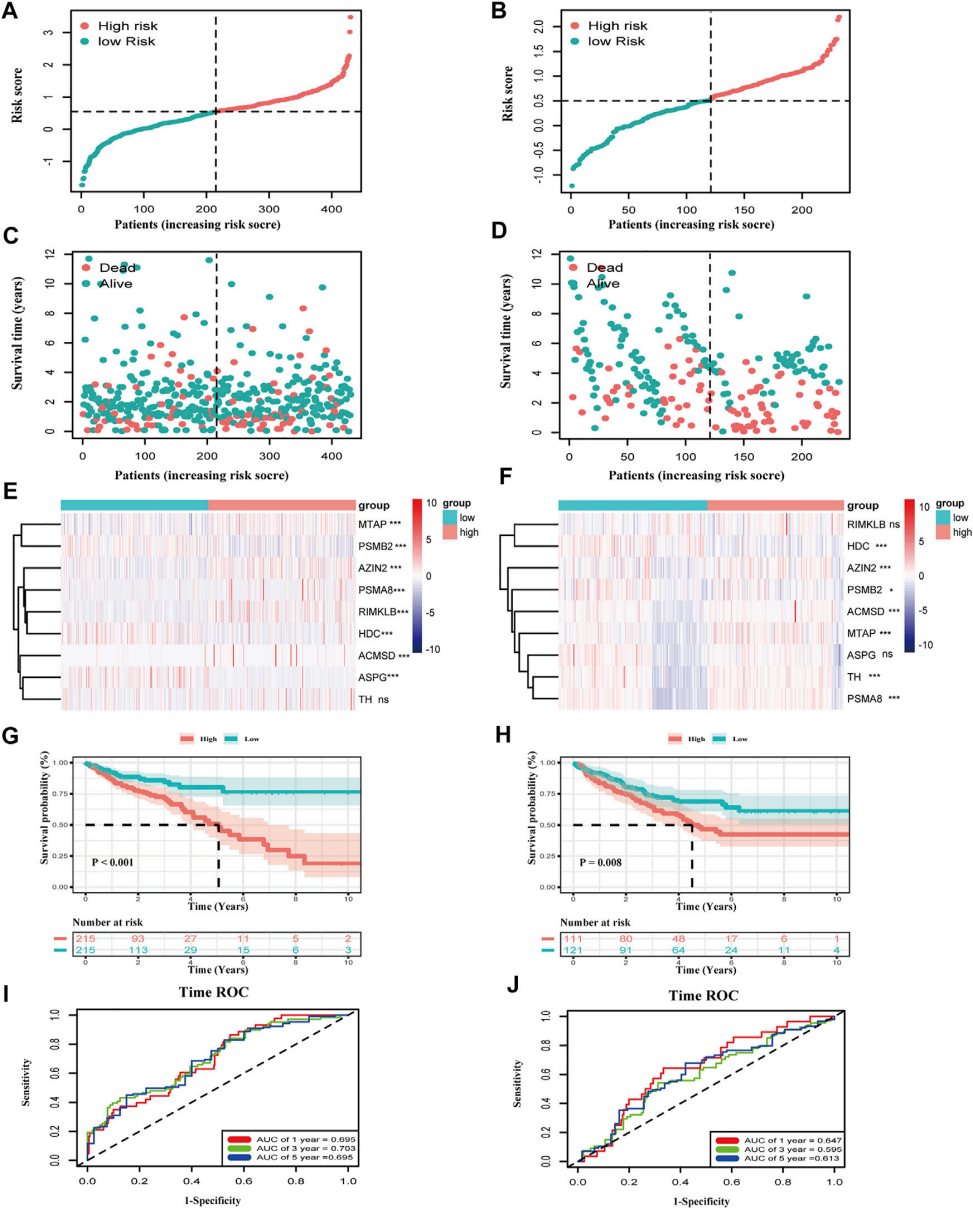
Validation of the risk model in TCGA-COAD and GSE17538. **(A,B)** The distribution of risk score in COAD patients. **(C,D)** Distribution of survival time in COAD patients. **(E,F)** Expression of the nine genes between the two groups in two datasets (***p < 0.001, **p < 0.01, *p < 0.05, ns: *p* ≥ 0.05). **(G,H)** K–M curves of the two groups in two datasets (TCGA-COAD: *p* < 0.001, GSE17539: *p* = 0.008). **(I,J)** Time ROC of 1, 3, and 5 survival years of COAD patients in the training and validation sets. The area under the curve is the AUC value (AUC of TCGA-COAD: 1 year = 0.695, 3 years = 0.703, and 5 years = 0.695; AUC of GSE17538: 1 year = 0.647, 3 years = 0.595, and 5 years = 0.613).

The evaluation of the risk model on the training and validation sets will be carried out in this part. First, we plotted the distribution of risk scores in the two cohorts: TCGA-COAD and GEO17538 (Figures 3A,B). The relationship between the patient’s survival time and survival status is shown in Figures 3C,D so that the survival information of each patient can be observed more intuitively. Subsequently, the expression of nine genes related to the amino acid metabolism in the two divided groups was exhibited with a heatmap. The expression of RIMKLB, HDC, and ASPG was relatively significant in the two groups of the two cohorts (Figures 3E,F). Next, the KM curve of the patients and the ROC at 1, 3, and 5 years were drawn. The distribution of survival curves in both training and validation sets was consistent: the group scoring higher had a poorer prognosis, and the group scoring lower had longer survival (TCGA: HR = 0.41, CI: 0.27–0.64; GEO: HR = 0.57, CI: 0.38–0.87). The two groups exhibited a different OS (TCGA: *p* < 0.01; GEO: *p* = 0.008) (Figures 3G,H). The 1-, 3-, and 5-year ROC splines were plotted; as a result of that, the robustness of the prognostic model was further confirmed (AUC of TCGA: 1 year = 0.695, 2 years = 0.703, and 3 years = 0.695; AUC of GEO: 1 year = 0.647, 2 years = 0.595, and 3 years = 0.613) (Figures 3H,I).

### Associations Between Risk Model and Clinicopathological Variables

**FIGURE 4:**
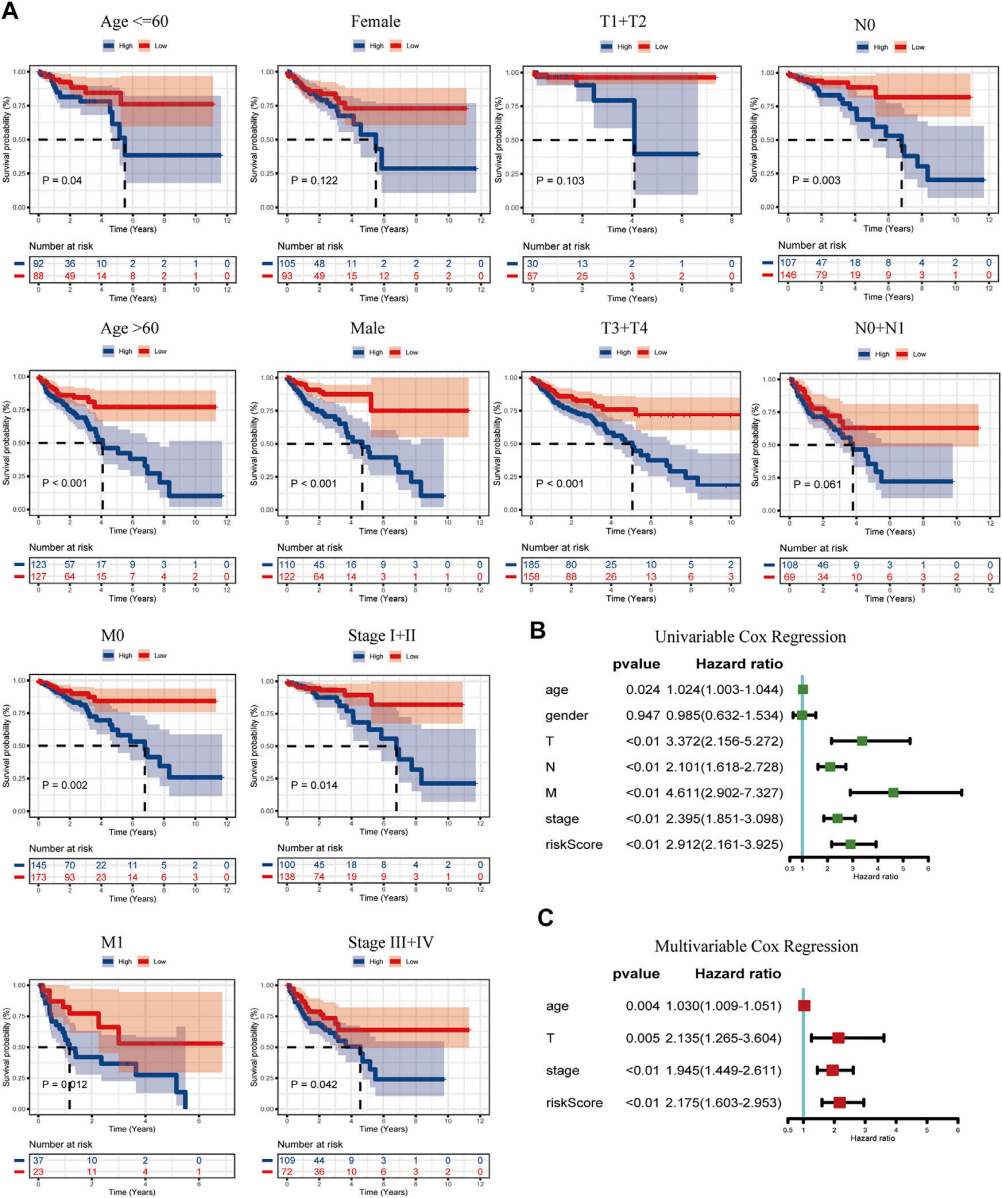
K–M curves of the risk model classified by clinicopathology features **(A)**, including age (≤65 years and >65 years), gender (female and male), T (T1+T2 and T3+T4), N (N0 and N1+N2), M (M0 and M1), and stages (I + II and III + IV). **(B)** Univariable Cox regression of the risk score linked with clinicopathology characters (age, gender, T, N, M, stage, and risk score). **(C)** Multivariable Cox regression of the risk score combined with the screened clinicopathology characters (age, T, stage, and risk score).

The discrepancy of risk scores by age, sex, pathological stage, and TNM stage of malignancy as defined by the AJCC is shown in Supplementary Figures S2A,B. Despite the indistinctive discrepancy in the risk score by age and sex, there were significant correlations in T, N, M, and stage (*p*-value: T: <0.01, N: <0.01, M: <0.01, stage: <0.01). The risk score increased with TNM and stage. Then, to verify whether the prognostic model would be different in subgroups with different clinical characteristics, KM curves grouped according to clinical information were drawn, including age ≤65 years vs. age>65 years, female vs. male, T1+T2 vs. T3+T4, N0 vs. N1+N2, M0 vs. N1, and Stage I + II vs. Stage III + IV (Figure 4A). Interestingly, no difference in age, M, and stage grouping was observed. In gender, the risk model had a higher accuracy of prediction for the outcome of male COAD patients (*p* < 0.01). For the T stage, the prognostic model was more suitable in COAD patients with a higher degree of invasion (*p* < 0.01). In the N stage, the N0 group is more suitable for the application of the prognostic model than the N1+N2 group. In conclusion, this prognostic risk model is more suitable for male COAD patients with a higher degree of local invasion but no lymph node metastasis. We then performed a univariate Cox regression on age, gender, T, N, M, stage, and risk score (Figure 4B), and it showed that gender was not a prognostic risk factor, but risk score along with age, TNM, and stage was qualified with a higher HR value (HR = 2.91, CI: 2.16–3.93, *p* < 0.01). Through the multivariate Cox analysis, clinically relevant variables were screened to age, T, stage, and risk score. The risk score still had a higher risk fold (HR = 2.18, CI: 1.60–2.95, *p* < 0.01), drawn by a forest plot (Figure 4C). This illustrated that the calculated score was equal to becoming an independent risk factor.

### Development of a Nomogram for Survival Prediction

**FIGURE 5:**
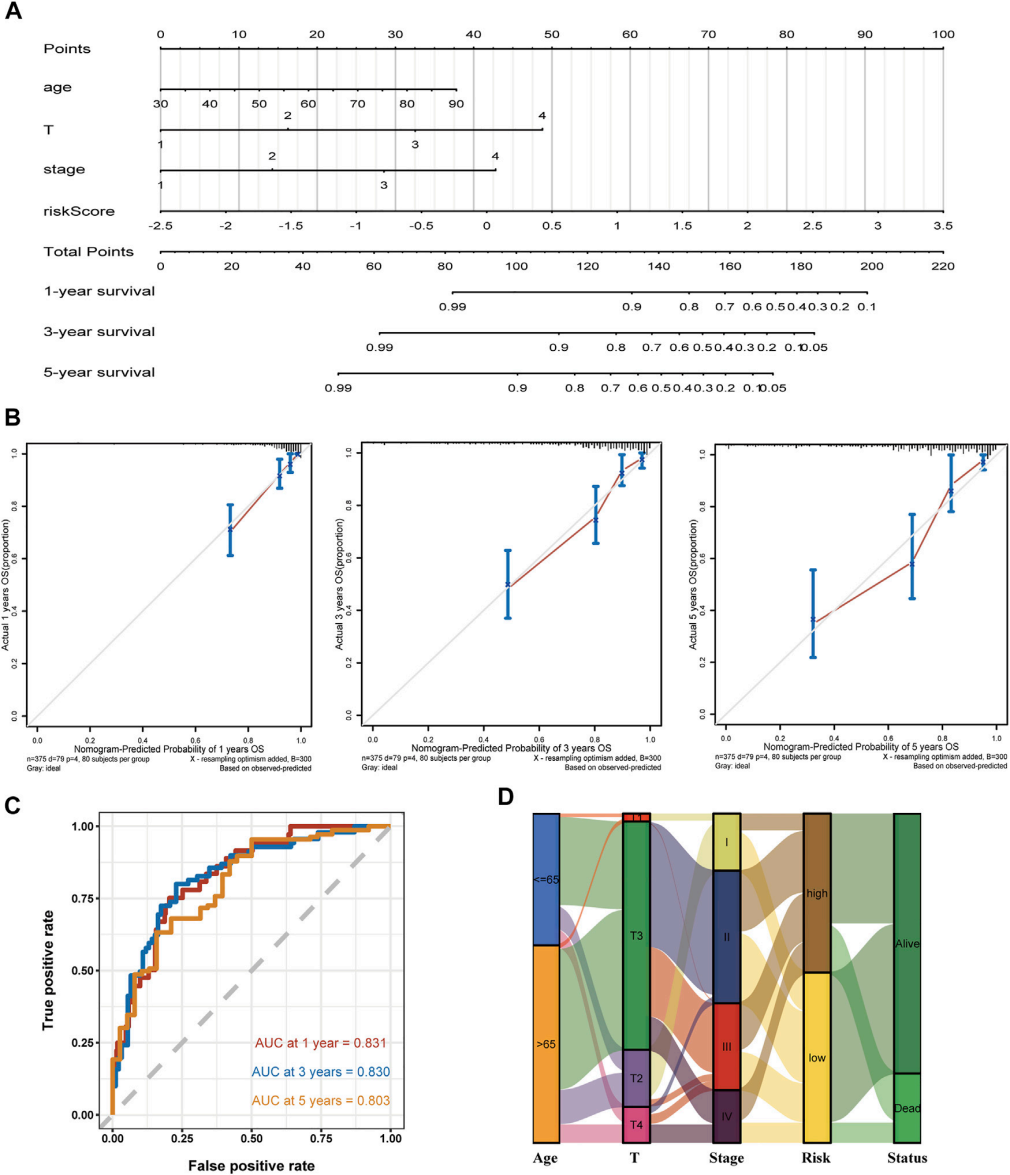
Establishment of a nomogram using the screened variable and the validation analysis. **(A,B)** The constructed nomogram and its correction curves. **(C)** AUC of the time ROC at 1, 3, and 5 years are 0.831, 0.830, and 0.803, respectively. **(D)** Alluvial plot shows the outcome of each COAD patient.

Compared with conventional clinical characteristics, the nomogram can directly use the graph to calculate the values of variables and then add them together, finally calculating the survival probability of the individual through the functional transformation relationship between the total points and the probability of the outcome event. Here we built a scoring system for each TCGA-COAD patient, which could calculate the total score according to the variables of age, T, stage, and risk score screened out by multivariate Cox regression, to predict patients’ OS of 1, 3, and 5 years (Figure 5A). In addition, to evaluate the precision of the nomogram predictions, calibration plots (Figure 5B) and ROC curves (Figure 5C) at 1, 3, and 5 years were drawn. The 1-, 3-, and 5-year AUC values all illustrated the credible specificity and sensitivity of the nomogram (AUC: 1 year = 0.831, 3 years = 0.830, and 5 years = 0.803). The alluvial plot visualized the shunting and OS of each COAD patient based on clinical features and group of risk score (Figure 5D). The DCA chart was used to evaluate the net benefit of the nomogram in predicting survival, demonstrating the high clinical efficacy of the constructed nomogram (Supplementary Figure S2C).

### The Enrichment of Immune-Related Pathways and the Relationship Between the Prognostic Risk Model and Immune Cell Infiltration

**FIGURE 6:**
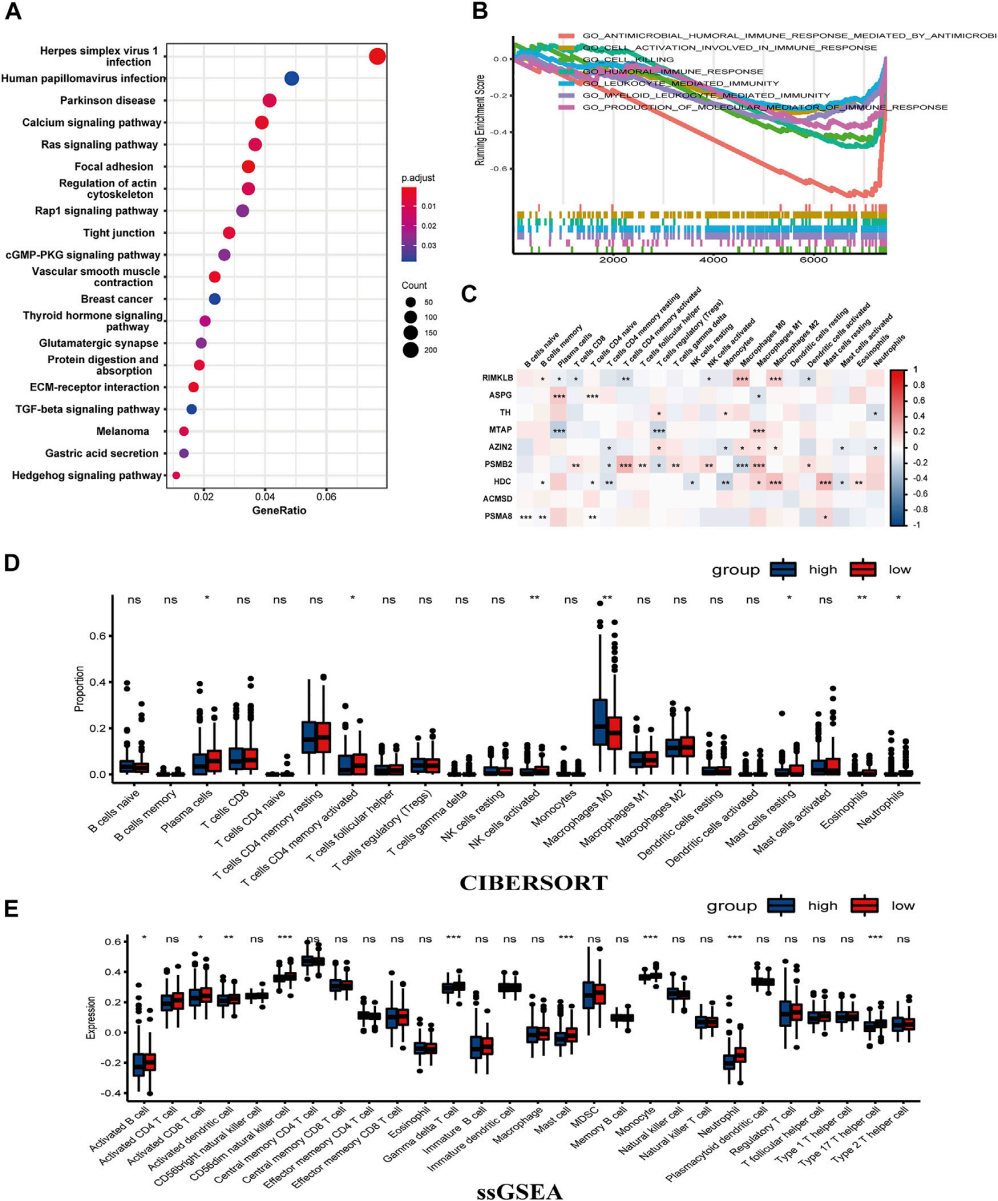
Enrichment analysis using DEGs of the two groups showed an association with immunity. **(A)** A dot chart of the KEGG. **(B)** GSEA illustrated that there was a connection between low-risk group and the tumor immunity. **(C)** The correlation between the nine genes and immune cells. **(D,E)** CIBERSORT and ssGSEA verified the connection (***p < 0.001, **p < 0.01, *p < 0.05, ns: *p* ≥ 0.05).

Differential genes between the classified two groups of TCGA-COAD were used for GO analysis, KEGG analysis, and GSEA. GO enriched 505 pathways, including external encapsulating structure organization, mitochondrial inner membrane, tubulin binding most significantly enriched in biological process (BP), cell component (CC), and molecular function (MF) (Supplementary Figure S3A; Supplementary Table S3). Meanwhile, 334 pathways were also enriched in KEGG, of which Herpes simplex virus 1 infection was the most significant (Supplementary Table S4). Interestingly, the group scoring higher was related to the calcium, Ras, Rap1, and TGF-beta signaling pathway (Figure 6A). Subsequently, 667 pathways were enriched by GSEA (|NES| >1, p-value <0.05, q-value <0.25) (Supplementary Table S5). Particularly, the low group was associated with the cell killing risk pathway, production of molecular mediator of immune response pathway, myeloid leukocyte-mediated immunity pathway, leukocyte-mediated immunity pathway, humoral immune response pathway, cell activation involved in the immune response pathway, and antimicrobial humoral immune response mediated by the antimicrobial peptide pathway (Figure 6B). This illustrated that our risk model was tightly linked to the immune of COAD. Then, ESTIMATE was applied to assess the tumor microenvironment (TME). In StromalScore and ESTIMATEScore, the scores between the two risk groups were not significant (*p* > 0.05). On the contrary, it was relatively significant in ImmuneScore (*p* = 0.057), with a higher trend seen in the low-risk group (Supplementary Figure S3B). To further estimate the association between the proportion of immune cells and the two groups, we utilized “Cibersort” to calculate tumor immune cell infiltration proportionally. In higher risk samples, the proportion of Macrophages M0 was higher. In lower risk samples, plasma cells, T cells, CD4 memory activated cells, NK cell activated, mast cell resting, eosinophils, and neutrophils are more aggregated (Figure 6C). An immune infiltration rainbow diagram of 430 patients is presented in Supplementary Figure S3C, with the horizontal axis representing samples, the left half representing the low-risk group, and the right half on behalf of the high-risk group. The vertical axis was the proportion of immune cell infiltration. The figure vividly exhibited the aggregation of immune cells in each patient. Meanwhile, the correlation analysis of nine genes related to the amino acid metabolism and 22 types of immune cells was carried out, the results of RILKMB, AZIN2, PSMB2, and HDC were more associated with these immune cells (Figure 6D). ssGSEA showed that compared to samples with a higher risk score, there existed significant differences in the expression of activated CD8 T cells, activated B cells, activated dendritic cells, CD56dim natural killer cell, gamma delta T cell, mast cell, monocyte, neutrophil, and type 17 T helper cell in samples with lower scores (Figure 6E). In conclusion, the group scoring lower showed a stronger immune infiltration response than the group scoring higher. Paclitaxel and Shikonin are common chemotherapeutic drugs screened with significant differences. Paclitaxel has a higher sensitivity in the high-risk group, while Shikonin has a higher sensitivity in the low-risk group (Supplementary Figure S4).

### Identification of Molecular Subtypes Employing the Amino Acid Metabolism-Related Nine Genes

**FIGURE 7:**
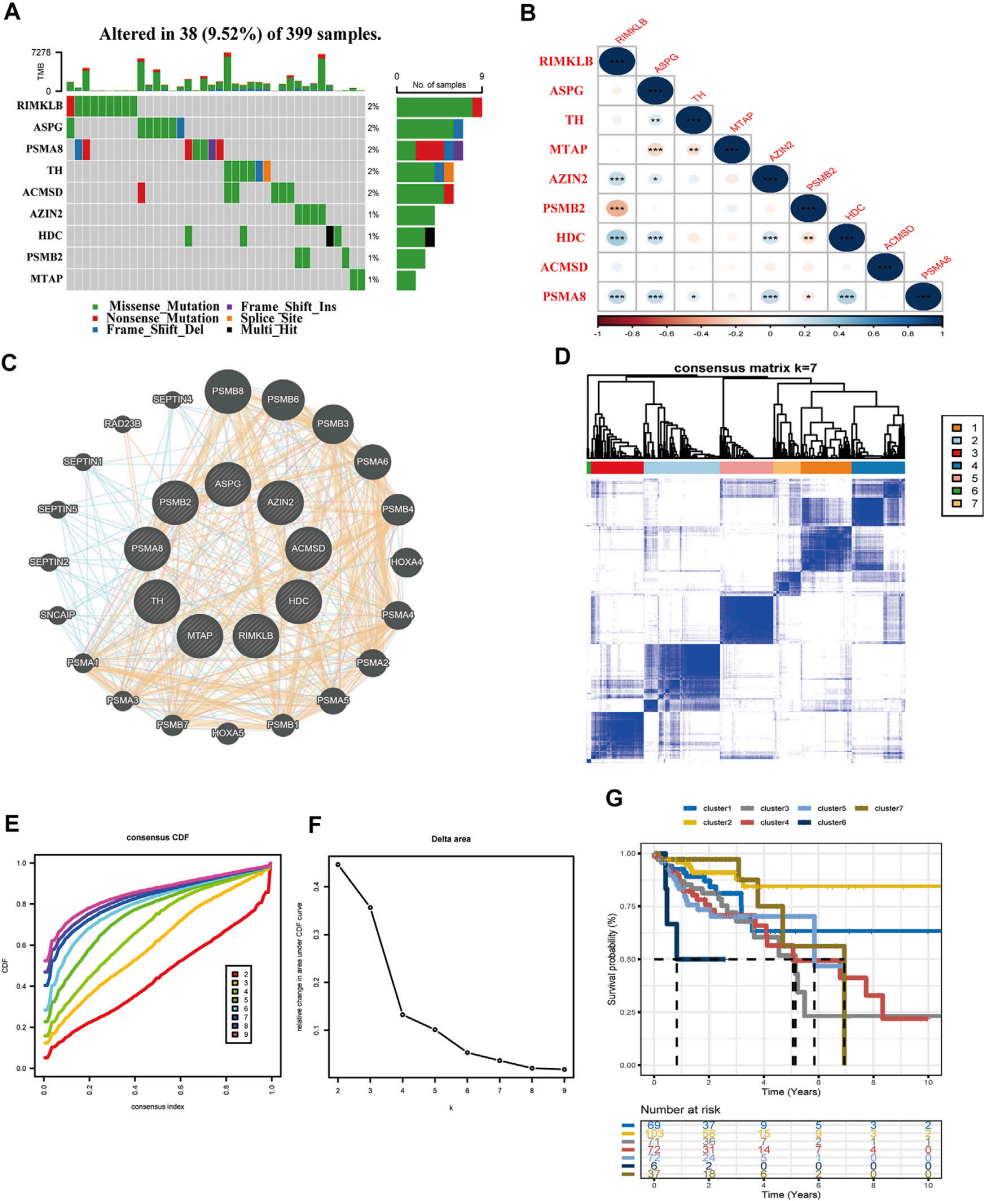
Interrelationships of nine genes involved in the amino acid metabolism. **(A)** TMB analysis of the nine genes in 399 samples of TCGA-COAD. **(B)** Spearman correlation analysis of the nine genes (***p < 0.001, **p < 0.01, *p < 0.05). **(C)** Protein interaction network analysis. **(D–F)** Consensus cluster analysis divides the gene signature into seven categories. **(G)** Validation of the seven clusters in the K–M plot.

This part presented the relationship between the nine amino acid metabolism-related genes and TMB, as well as the connection and interaction between the nine genes, and finally used consensus clustering to determine whether these nine genes could divide patients into different molecular subtypes. First of all, TMB analysis showed that the mutation frequency of RIMKLB, ASPG, PSMA8, and TH was higher than that of the remaining genes in 399 samples. It is worth mentioning that RIMKLB also has a certain degree of correlation with immune cells, which means that this gene may be used as an immune drug target (Figure 7A). The Spearman correlation coefficients of the nine genes are presented in Figure 7B. RIMKLB, HDC, and AZIN share a significant positive correlation (*p* < 0.001; Figure 7B). Through the PPI network analysis, AZIN2 correlated with four other genes, which may function as a hub gene. Meanwhile, PSMA8 was strongly associated with PSMB2 (Figure 7C). Finally, consensus clustering analyses were performed to form different molecular subtypes. The results suggested considerably seven categories (Figures 7D–F). Based on survival analysis, molecular subtypes exhibited various OS, which well demonstrated the possible biological contribution of molecular subtype classification methods (Figure 7G). The analysis also showed that the selected nine genes have a high potential to be biomarkers for COAD, demonstrating that the amino acid metabolism-related genes may possess a part to play in clinical contribution.

### Hub Genes Associated With Immunity and Amino Acid Metabolism Identified in Weighted Gene Co-Expression Network Analysis

**FIGURE 8:**
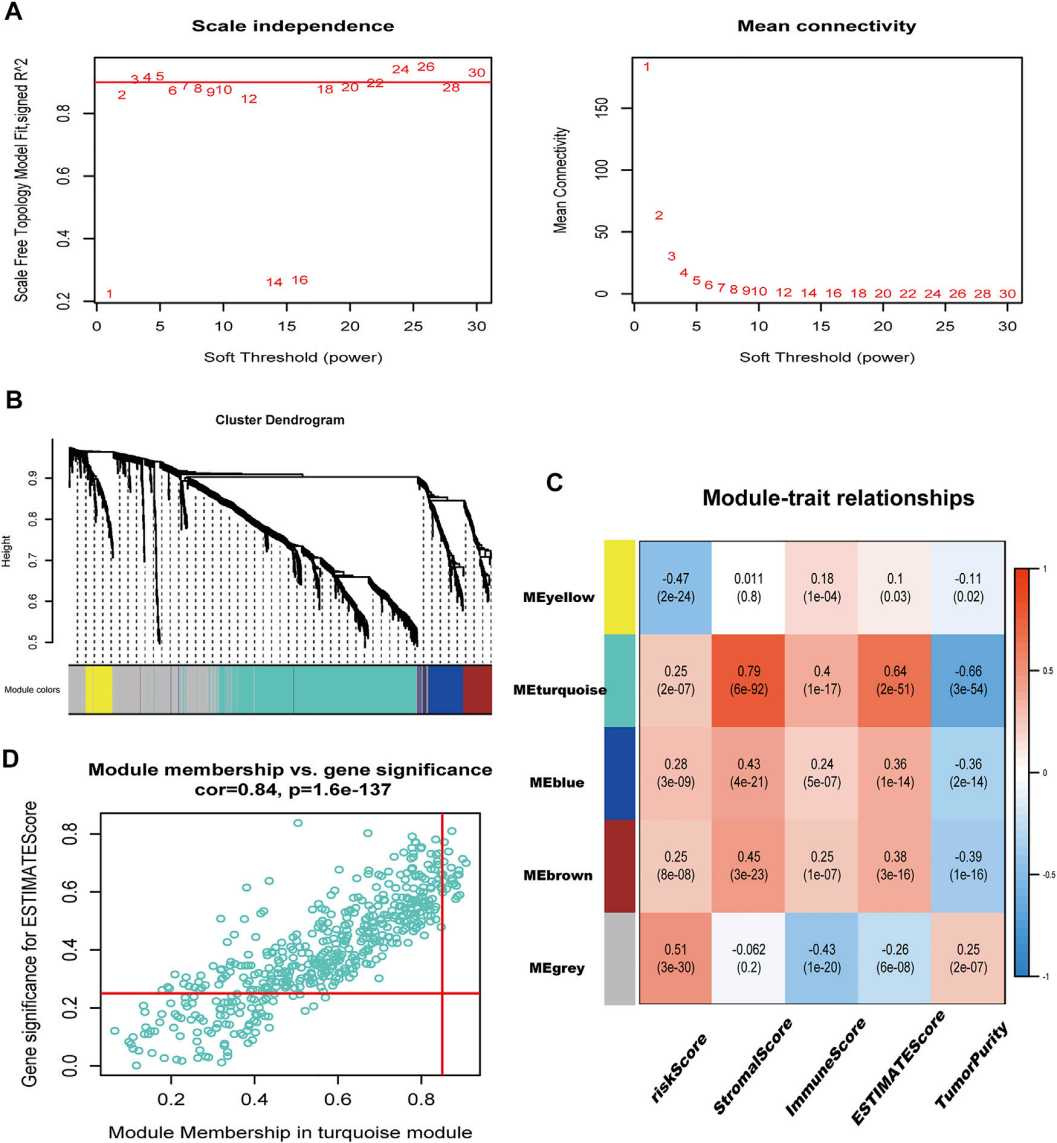
immune-related genes were identified in WGCNA. **(A)** Filtering of soft power. **(B)** Diagram of five modules with different colors. **(C)** Heatmap of the correlation between modules and immune-related scores. **(D)** Identification of genes with high GS and MM (GS > 0.25, MM > 0.85).

A total of 1283 DEGs were yielded between the two risk groups. The modules related to immune-related score were obtained by WGCNA (Figures 8A–C). Correlation between the Turquoise module and ESTIMATE score (R = 0.64, P = 2E-51), and risk score (R = 0.25, P = 2E-07) is shown in Figure 8C. After that, we obtained 22 hub genes in the Turquoise module (PRELP, PLN, AOC3, COL8A1, MPDZ, STON1, LMOD1, RNF150, MSRB3, CACNA2D1, NAP1L3, BNC2, SGCD, FNDC1, HSPB8, FBN1, CCDC80, TNS1, MYLK, DDR2, MAP1A, and BOC) using a criterion of MM > 0.85 and GS > 0.25.

## Discussion

Metabolism reprogramming is considered a hallmark of cancer. This biochemical process is ruled by oncogenic and tumor suppressor genes, which offered energy, reducing equivalents and biosynthetic precursors for tumors (Vander Heiden and DeBerardinis, 2017). Amino acid metabolism, taking part in metabolism reprogramming, proves to participate in the proliferation of cancer (Li and Zhang, 2016; Vettore et al., 2020). Glutamine, serine, and glycine, which are recently being focused on, act as raw materials for protein synthesis as well as metabolic regulators to control cell development (Li and Zhang, 2016). Recoding of the amino acid metabolism also applies to colorectal cancer. In CRC with mutated KRAS, the upregulation of ASNS enables the tumor to adapt to high glutamine demands (Toda et al., 2016). In addition, PKCζ can negatively regulate serine–glycine biosynthesis in colorectal cancer in the absence of glucose, thereby promoting intestinal tumorigenesis in *Apcmin* mice (Ma et al., 2013). CRC occupies a third in incidence and mortality worldwide. Although its mortality rates are decreasing, the decline has slowed down in recent years (Sung et al., 2021). It is worthy to mention that the 5-year survival rate with distant advanced colon cancer is down to 14%, which indicates that how to evaluate the prognosis of COAD has become one of the key points (Mattiuzzi et al., 2019). Given the valuable potential that the amino acid metabolism demonstrates in COAD, we built a prognosis risk model for assessing COAD patients’ outcomes.

Our study collected RNA-seq expression profiles of TCGA-COAD as well as GEO17538 and corresponding clinical data. Through the GSEA website, we acquired genes related to the amino acid metabolism, applying them to obtain the intersection of three gene sets. Then, univariable and multivariable Cox regressions, as well as LASSO analysis, were applied to select genes concerning prognosis. Finally, nine prognostic related genes were obtained to establish a risk model. Among the nine genes, ribosomal modification protein rimK like family member B (RIMKLB) is a protein-coding gene involved in the glutamine family amino acid metabolic process and cellular protein modification process, which is proved to be associated with prognosis and clinical stage (Li et al., 2021). Asparaginase (ASPG) is known to be related to asparagine degradation. A study discovered that asparaginase is highly noxious to CRC with WNT-activating mutations inhibiting GSK3 (Hinze et al., 2020). Tyrosine hydroxylase (TH), a rate-limiting enzyme in the synthesis of catecholamines, is possible to be down-regulated in COAD, which may affect the neural integrity of the perivascular plexus (Chamary et al., 2000). As the first step of the salvage pathway for methionine, methylthioadenosine phosphorylase (MTAP) accelerates the proliferation and metastasis of CRC through epithelial–mesenchymal transition (EMT) (Chamary et al., 2000). Antizyme inhibitor 2 (AZIN2) takes part in the ornithine metabolic process and is identified to be an element for a poor prognosis in CRC, actuating aggressiveness of cancer cells with morphological characters of EMT (Kaprio et al., 2019). Proteasome 20S subunit alpha 8 (PSMA8) is predicted to participate in the meiotic cell cycle and proteasomal protein catabolic process, and its genetic variants are identified to link with the survival of CRC (Jiao et al., 2018). Histidine decarboxylase (HDC), which plays a role in the process of histidine catabolic, correlates with CRC stage and blood supply (Masini et al., 2005). Moreover, HDC-expressing granulocyte myeloid cell subsets regulate CD8 T cells by the regulation of Tregs and therefore are of vital importance in suppressing tumoricidal immunity (Chen et al., 2017). In summary, our study corroborated again that RIMKLB, TH, MTAP, AZIN2, HDC, and PSMA8 are directly or indirectly associated with the progression and prognosis of CRC. However, research on proteasome 20S subunit beta 2 (PSMB2) and aminocarboxymuconate semi-aldehyde decarboxylase (ACMSD) are waiting to be explored in bowel cancer.

We then utilized the prognostic risk model acquired by the former analysis to divide patients into two groups utilizing the median risk score. The OS of the two groups was evaluated, and as a result of that, the prognosis of the two showed significant discrepancy (*p* < 0.0001). This difference was verified in GEO17538, which proved that to a certain degree, the amino acid metabolism partakes in the prognosis of COAD patients. With analyses related to clinicopathological features further conducted, we concluded that risk scores derived from genes associated with the amino acid metabolism can become a measure of COAD patient prognosis. The model is suitable for male patients with higher T stage but no metastasis of lymph node (Figure 4A), which is more convincing than using one single gene as a grouping criterion to estimate the outcome. To better evaluate the adaptability and scientificity of the risk model, we compared our research with other prognostic signatures of the amino acid metabolism in different cancers. In hepatocellular carcinoma, the identification of amino acid catabolism-related genes was performed to construct a risk model (Zhao et al., 2021). They applied LASSO analysis, built a nomogram combined with clinical data, put functional enrichment analysis into practice, and added experiments in validating genes’ expression. However, immune cell infiltration analysis they explored had not been conducted more in-depth. In osteosarcoma, Wan et al. created a risk model for osteosarcoma using glutamine metabolism-related genes. While immune microenvironment analysis was still waiting to be verified (Wan et al., 2022), Jiang et al. investigated genes related to branched-chain amino acid (BCAA) metabolism in pancreatic cancer. They constructed the risk model by the univariable Cox regression and LASSO analysis while lacking definite specificity and sensitivity tests and relatively convincing immune cell analysis (Jiang et al., 2022). Therefore, combining the methods and results of these studies, we explored the prospect of our prognostic model in clinical applications, corresponding immune infiltration analysis, which may exhibit the close contact between amino acid metabolism and tumor microenvironment.

Cell proliferation, continued growth, and avoidance of cell death are hallmarks of cancer, which require massive energy (Hanahan and Weinberg, 2011). Growing evidence suggests that interactions between immune cells and metabolites might be significant in regulating immunity and tumor immunoevasion (DePeaux and Delgoffe, 2021). Meanwhile, the screening of immune targets plays a key role in the immunotherapy of MSI-H colorectal cancer (Liu J. et al., 2021; Liu et al., 2022). In our study, the group scoring lower had more immune cell infiltration, the cause of which can be explained as follows. Glutamine acts as a principal amino acid for energy generation and functions as a metabolic intermediate. A study shows that the blockage of glutamine in mice with colon cancer inhibits the metabolic process of tumor cells in oxygen and glucose. Oppositely, effector T cells adapt to glutamine antagonism by altering their oxygen metabolism for a long-live, more activated phenotype (Leone et al., 2019). RIMKLB participates in the glutamine family amino acid metabolic process. The low-risk group had a relatively low expression of RIMKLB but achieved a stronger T-cell infiltration (Figure 6E), which justified the lack of glutamate and the self-adaptation of T cells. In addition, TMB analysis revealed that RIMKLB may have the potential to be an immune target (Figure 7A). MTAP is involved in methionine catabolism. Methionine is required for T-cell differentiation, and a reduction in methionine results in the decrease of the level of epigenetic methyl donor S-adenosyl-l-methionine (Roy et al., 2020). Studies demonstrate that CD8^+^ T cells isolated from methionine-deficient tumors are also deficient in S-adenosyl-l-methionine, leading to lower expression of STAT5, a signaling pathway that is essential for T cells’ response to IL-7 and IL-15 (Tripathi et al., 2010). Colorectal tumor cells are discovered to contend with T cells for methionine simultaneously in TME (DePeaux and Delgoffe, 2021). Our study revealed that the group scoring higher had more expression of MTAP, which indicated a shortage of methionine. It is consistent with the depletion of methionine due to the massive demand for tumor cells and T cells. Meanwhile, this matches the result of the disruption of the T-cell methionine metabolic pathway by tumor cells (Bian et al., 2020). Furthermore, PSMA8 predicts favorable outcomes in cancer and is associated with immune response signaling, which corresponded with our study (Chiao et al., 2021). In addition, PSMB2 shares the same conclusion with PSMA8. ACMSD ultimately regulates the metabolic outcome of tryptophan (Trp) catabolism. Depletion of Trp and Trp-Kyn-AHR-related metabolism results in cancer immunity evasion (Wang and Zou, 2020), which shares consistency with our study. L-asparaginase (ASPG) is used to treat acute lymphoblastic leukemia (Couturier et al., 2015; Touzart et al., 2019), where a significant up-regulated relationship with the low-risk group is observed. The massive immune infiltration of the low-risk group indicated an association between ASPG and TME, which is worthwhile exploring. In conclusion, our research established that a prognostic marker of amino acid metabolism is closely linked to the tumor immune microenvironment. The signature provides guidance for the evaluation of the survival of patients along with the direction and targets of treatment. PSMB2 and ACMSD were newly identified in COAD as prognostic related genes, the mechanism of which can be further explored.

There are some limitations to our study. First, we used public databases for analysis. Therefore, the genes related to the amino acid metabolism had not been verified *in vitro* experiments. The underlying mechanisms associated with immune regulation have not been elucidated. In addition, as a retrospective study, there is some potential bias compared to prospective studies. Research with more COAD patients should be conducted for further validation.

## Conclusion

In summary, we identified nine novel amino acid metabolism-related gene signatures in COAD. Then a risk model was built and combined with clinical features. Furthermore, we discussed the relationship between the model and tumor immunity. While more samples should be included to increase credibility, a deeper mechanism should be explored.

## Data Availability

The original contributions presented in the study are included in the article/Supplementary Material; further inquiries can be directed to the corresponding author.

## Supplementary Material

Supplementary Figure S1 GO analysis and expression of nine genes in tumor and cancer tissues. **(A)** GO enrichment analysis of DEGs of the TCGA-COAD, revealing that amino acid metabolism partakes in COAD development. **(B)** The expression of the nine screened genes in tumor and healthy tissues showed a significant difference.

Supplementary Figure S2 The relationship between the risk score and clinical features and DCA plot of the nomogram. **(A,B)** The relationship between risk score and clinical characters. T, N, M, and stage were in positive correlation with the risk score. **(C)** DCA of the nomogram exhibited a robust nomogram.

Supplementary Figure S3 GO analysis in risk model and ESTIMATE along with the rainbow figure of CIBERSORT. **(A)** GO plot of the DEGs of risk groupings. **(B)** ESTIMATE of the risk model, which revealed a relatively meaningful outcome in ImmuneScore (*p* = 0.057). **(C)** The distribution of immune cells in 399 samples.

Supplementary Figure S4 IC50 analysis were calculated. **(A)** Relationship between paclitaxel and risk score. **(B)** Relationship between Shikonin and risk score.

Supplementary Table S1 1353 GO analysis between tumor and normal tissues of colon cancer in TCGA.

Supplementary Table S2 Univariable and multivariable COX regressions of amino acid metabolism-related genes. Nine genes were selected to construct the prognostic model (HR: hazard ratio; HR.95L and HR.95H: 95% confidence interval).

Supplementary Table S3 505 GO pathways obtained between high- and low-risk groups.

Supplementary Table S4 334 KEGG pathways obtained between high- and low-risk groups.

Supplementary Table S5 667 GSEA pathways obtained between high- and low-risk groups.

## Abbreviations

AJCC, American Joint Committee on Cancer; AUC, area under the respective ROC; BCAA, branched-chain amino acids; COAD, colon adenocarcinoma; CRC, colorectal cancer; DCA, decision curve analysis; DEGs, differentially expressed genes; GO, gene ontology; GSEA, gene set enrichment analysis; HR, hazard ratio; KEGG, Kyoto Encyclopedia of Genes and Genomes; K–M, Kaplan–Meier; LASSO, least absolute shrinkage and selection operator; OS, overall survival; PPI, protein–protein interaction; ROC, receiver operating characteristic; ROS, reactive oxygen species; ssGSEA, single sample gene set enrichment analysis; TMB, tumor mutation burden; WGCNA, weighted gene co-expression network analysis.

## References

[B1] Bian Y. Li W. Kremer D. M. Sajjakulnukit P. Li S. Crespo J. (2020). Cancer SLC43A2 Alters T Cell Methionine Metabolism and Histone Methylation. Nature 585 (7824), 277–282. 10.1038/s41586-020-2682-1 32879489 PMC7486248

[B2] Biller L. H. Schrag D. (2021). Diagnosis and Treatment of Metastatic Colorectal Cancer: A Review. J. Am. Med. Assoc. 325 (7), 669–685. 10.1001/jama.2021.0106 33591350

[B3] Boroughs L. K. DeBerardinis R. J. (2015). Metabolic Pathways Promoting Cancer Cell Survival and Growth. Nat. Cell Biol. 17 (4), 351–359. 10.1038/ncb3124 25774832 PMC4939711

[B4] Chamary V. Robson T. Loizidou M. Boulos P. Burnstock G. (2000). Progressive Loss of Perivascular Nerves Adjacent Tocolorectal Cancer. Eur. J. Surg. Oncol. (EJSO) 26 (6), 588–593. 10.1053/ejso.2000.0952 11034811

[B5] Chen X. Takemoto Y. Deng H. Middelhoff M. Friedman R. A. Chu T. H. (2017). Histidine Decarboxylase (HDC)-expressing Granulocytic Myeloid Cells Induce and Recruit Foxp3(+) Regulatory T Cells in Murine Colon Cancer. Oncoimmunology 6 (3), e1290034. 10.1080/2162402x.2017.1290034 28405523 PMC5384347

[B6] Chiao C. C. Liu Y. H. Phan N. N. An Ton N. T. Ta H. D. K. Anuraga G. (2021). Prognostic and Genomic Analysis of Proteasome 20S Subunit Alpha (PSMA) Family Members in Breast Cancer. Diagn. (Basel) 11 (12), 2220. 10.3390/diagnostics11122220 PMC8699889 34943457

[B7] Cohen A. S. Geng L. Zhao P. Fu A. Schulte M. L. Graves-Deal R. (2020). Combined Blockade of EGFR and Glutamine Metabolism in Preclinical Models of Colorectal Cancer. Transl. Oncol. 13 (10), 100828. 10.1016/j.tranon.2020.100828 32652471 PMC7348062

[B8] Couturier M.-A. Huguet F. Chevallier P. Suarez F. Thomas X. Escoffre-Barbe M. (2015). Cerebral Venous Thrombosis in Adult Patients with Acute Lymphoblastic Leukemia or Lymphoblastic Lymphoma during Induction Chemotherapy Withl-Asparaginase: The GRAALL Experience. Am. J. Hematol. 90 (11), 986–991. 10.1002/ajh.24130 26214580

[B9] DePeaux K. Delgoffe G. M. (2021). Metabolic Barriers to Cancer Immunotherapy. Nat. Rev. Immunol. 21 (12), 785–797. 10.1038/s41577-021-00541-y 33927375 PMC8553800

[B10] Favoriti P. Carbone G. Greco M. Pirozzi F. Pirozzi R. E. M. Corcione F. (2016). Worldwide Burden of Colorectal Cancer: a Review. Updat. Surg. 68 (1), 7–11. 10.1007/s13304-016-0359-y 27067591

[B11] Galon J. Costes A. Sanchez-Cabo F. Kirilovsky A. Mlecnik B. Lagorce-Pagès C. (2006). Type, Density, and Location of Immune Cells within Human Colorectal Tumors Predict Clinical Outcome. Science 313 (5795), 1960–1964. 10.1126/science.1129139 17008531

[B12] Geng Q. Wei Q. Shen Z. Zheng Y. Wang L. Xue W. (2021). Comprehensive Analysis of the Prognostic Value and Immune Infiltrates of the Three-m5C Signature in Colon Carcinoma. Cancer. Manag. Res. 13, 7989–8002. 10.2147/cmar.s331549 34707405 PMC8542737

[B13] Hanahan D. Weinberg R. A. (2011). Hallmarks of Cancer: the Next Generation. Cell 144 (5), 646–674. 10.1016/j.cell.2011.02.013 21376230

[B14] Hinze L. Labrosse R. Degar J. Han T. Schatoff E. M. Schreek S. (2020). Exploiting the Therapeutic Interaction of WNT Pathway Activation and Asparaginase for Colorectal Cancer Therapy. Cancer Discov. 10 (11), 1690–1705. 10.1158/2159-8290.cd-19-1472 32703769 PMC7642035

[B15] Jahanafrooz Z. Mosafer J. Akbari M. Hashemzaei M. Mokhtarzadeh A. Baradaran B. (2020). Colon Cancer Therapy by Focusing on Colon Cancer Stem Cells and Their Tumor Microenvironment. J. Cell Physiol. 235 (5), 4153–4166. 10.1002/jcp.29337 31647128

[B16] Jiang Z. Zheng J. Liu J. Yang X. Chen K. (2022). Novel Branched-Chain Amino Acid-Catabolism Related Gene Signature for Overall Survival Prediction of Pancreatic Carcinoma. J. Proteome Res. 21 (3), 740–746. 10.1021/acs.jproteome.1c00607 34816714

[B17] Jiao X. Liu W. Mahdessian H. Bryant P. Ringdahl J. Timofeeva M. (2018). Recurrent, Low-Frequency Coding Variants Contributing to Colorectal Cancer in the Swedish Population. PLoS One 13 (3), e0193547. 10.1371/journal.pone.0193547 29547645 PMC5856271

[B18] Kaprio T. Rasila T. Hagström J. Mustonen H. Lankila P. Haglund C. (2019). Ornithine Decarboxylase Antizyme Inhibitor 2 (AZIN2) Is a Signature of Secretory Phenotype and Independent Predictor of Adverse Prognosis in Colorectal Cancer. PLoS One 14 (2), e0211564. 10.1371/journal.pone.0211564 30768610 PMC6377119

[B19] Langfelder P. Horvath S. (2008). WGCNA: an R Package for Weighted Correlation Network Analysis. BMC Bioinforma. 9, 559. 10.1186/1471-2105-9-559 PMC2631488 19114008

[B20] Le Y. Zhang S. Ni J. You Y. Luo K. Yu Y. (2018). Sorting Nexin 10 Controls mTOR Activation through Regulating Amino-Acid Metabolism in Colorectal Cancer. Cell Death Dis. 9 (6), 666. 10.1038/s41419-018-0719-2 29867114 PMC5986761

[B21] Leone R. D. Zhao L. Englert J. M. Sun I.-M. Oh M.-H. Sun I.-H. (2019). Glutamine Blockade Induces Divergent Metabolic Programs to Overcome Tumor Immune Evasion. Science 366 (6468), 1013–1021. 10.1126/science.aav2588 31699883 PMC7023461

[B22] Li Z. Zhang H. (2016). Reprogramming of Glucose, Fatty Acid and Amino Acid Metabolism for Cancer Progression. Cell. Mol. Life Sci. 73 (2), 377–392. 10.1007/s00018-015-2070-4 26499846 PMC11108301

[B23] Li X. Wen D. Li X. Yao C. Chong W. Chen H. (2020). Identification of an Immune Signature Predicting Prognosis Risk and Lymphocyte Infiltration in Colon Cancer. Front. Immunol. 11, 1678. 10.3389/fimmu.2020.01678 33013820 PMC7497441

[B24] Li N. Guo Q. Zhang Q. Chen B. J. Li X. A. Zhou Y. (2021). Comprehensive Analysis of Differentially Expressed Profiles of mRNA N6-Methyladenosine in Colorectal Cancer. Front. Cell Dev. Biol. 9, 760912. 10.3389/fcell.2021.760912 35087827 PMC8787460

[B25] Lino-Silva L. S. Xinaxtle D. L. Salcedo-Hernández R. A. (2020). Tumor Deposits in Colorectal Cancer: the Need for a New "pN" Category. Ann. Transl. Med. 8 (12), 733. 10.21037/atm.2020.03.175 32647658 PMC7333091

[B26] Liu J. Geng R. Ni S. Cai L. Yang S. Shao F. (2022). Pyroptosis-related lncRNAs Are Potential Biomarkers for Predicting Prognoses and Immune Responses in Patients with UCEC. Mol. Ther. - Nucleic Acids 27, 1036–1055. 10.1016/j.omtn.2022.01.018 35228898 PMC8844853

[B27] Liu J. Geng R. Yang S. Shao F. Zhong Z. Yang M. (2021). Development and Clinical Validation of Novel 8-Gene Prognostic Signature Associated with the Proportion of Regulatory T Cells by Weighted Gene Co-Expression Network Analysis in Uterine Corpus Endometrial Carcinoma. Front. Immunol. 12, 788431. 10.3389/fimmu.2021.788431 34970268 PMC8712567

[B28] Liu X. Liu Y. Liu Z. Lin C. Meng F. Xu L. (2021). CircMYH9 Drives Colorectal Cancer Growth by Regulating Serine Metabolism and Redox Homeostasis in a P53-dependent Manner. Mol. Cancer 20 (1), 114. 10.1186/s12943-021-01412-9 34496888 PMC8424912

[B29] Ma L. Tao Y. Duran A. Llado V. Galvez A. Barger J. F. (2013). Control of Nutrient Stress-Induced Metabolic Reprogramming by PKCζ in Tumorigenesis. Cell 152 (3), 599–611. 10.1016/j.cell.2012.12.028 23374352 PMC3963830

[B30] Masini E. Fabbroni V. Giannini L. Vannacci A. Messerini L. Perna F. (2005). Histamine and Histidine Decarboxylase Up-Regulation in Colorectal Cancer: Correlation with Tumor Stage. Inflamm. Res. 54 Suppl 1 (Suppl. 1), S80–S81. 10.1007/s00011-004-0437-3 15928846

[B31] Mattiuzzi C. Sanchis-Gomar F. Lippi G. (2019). Concise Update on Colorectal Cancer Epidemiology. Ann. Transl. Med. 7 (21), 609. 10.21037/atm.2019.07.91 32047770 PMC7011596

[B32] Mejri N. Dridi M. El Benna H. Labidi S. Daoud N. Boussen H. (2018). Tumor Location Impact in Stage II and III Colon Cancer: Epidemiological and Outcome Evaluation. J. Gastrointest. Oncol. 9 (2), 263–268. 10.21037/jgo.2017.12.02 29755764 PMC5934145

[B33] Mlecnik B. Tosolini M. Kirilovsky A. Berger A. Bindea G. Meatchi T. (2011). Histopathologic-based Prognostic Factors of Colorectal Cancers Are Associated with the State of the Local Immune Reaction. J. Clin. Oncol. 29 (6), 610–618. 10.1200/jco.2010.30.5425 21245428

[B34] Roy D. G. Chen J. Mamane V. Ma E. H. Muhire B. M. Sheldon R. D. (2020). Methionine Metabolism Shapes T Helper Cell Responses through Regulation of Epigenetic Reprogramming. Cell Metab. 31 (2), 250–266. 10.1016/j.cmet.2020.01.006 32023446

[B35] Siegel R. L. Miller K. D. Goding Sauer A. Fedewa S. A. Butterly L. F. Anderson J. C. (2020). Colorectal Cancer Statistics, 2020. CA A Cancer J. Clin. 70 (3), 145–164. 10.3322/caac.21601 32133645

[B36] Subramanian A. Tamayo P. Mootha V. K. Mukherjee S. Ebert B. L. Gillette M. A. (2005). Gene Set Enrichment Analysis: a Knowledge-Based Approach for Interpreting Genome-wide Expression Profiles. Proc. Natl. Acad. Sci. U.S.A. 102 (43), 15545–15550. 10.1073/pnas.0506580102 16199517 PMC1239896

[B37] Sung H. Ferlay J. Siegel R. L. Laversanne M. Soerjomataram I. Jemal A. (2021). Global Cancer Statistics 2020: GLOBOCAN Estimates of Incidence and Mortality Worldwide for 36 Cancers in 185 Countries. CA Cancer J. Clin. 71 (3), 209–249. 10.3322/caac.21660 33538338

[B38] Tardito S. Oudin A. Ahmed S. U. Fack F. Keunen O. Zheng L. (2015). Glutamine Synthetase Activity Fuels Nucleotide Biosynthesis and Supports Growth of Glutamine-Restricted Glioblastoma. Nat. Cell Biol. 17 (12), 1556–1568. 10.1038/ncb3272 26595383 PMC4663685

[B39] Toda K. Kawada K. Iwamoto M. Inamoto S. Sasazuki T. Shirasawa S. (2016). Metabolic Alterations Caused by KRAS Mutations in Colorectal Cancer Contribute to Cell Adaptation to Glutamine Depletion by Upregulation of Asparagine Synthetase. Neoplasia 18 (11), 654–665. 10.1016/j.neo.2016.09.004 27764698 PMC5071549

[B40] Touzart A. Lengliné E. Latiri M. Belhocine M. Smith C. Thomas X. (2019). Epigenetic Silencing Affects L-Asparaginase Sensitivity and Predicts Outcome in T-ALL. Clin. Cancer Res. 25 (8), 2483–2493. 10.1158/1078-0432.ccr-18-1844 30659025

[B41] Tripathi P. Kurtulus S. Wojciechowski S. Sholl A. Hoebe K. Morris S. C. (2010). STAT5 Is Critical to Maintain Effector CD8+T Cell Responses. J. Immunol. 185 (4), 2116–2124. 10.4049/jimmunol.1000842 20644163 PMC2991082

[B42] Vander Heiden M. G. DeBerardinis R. J. (2017). Understanding the Intersections between Metabolism and Cancer Biology. Cell 168 (4), 657–669. 10.1016/j.cell.2016.12.039 28187287 PMC5329766

[B43] Vettore L. Westbrook R. L. Tennant D. A. (2020). New Aspects of Amino Acid Metabolism in Cancer. Br. J. Cancer 122 (2), 150–156. 10.1038/s41416-019-0620-5 31819187 PMC7052246

[B44] Wan L. Zhang W. Liu Z. Yang Z. Tu C. Li Z. (2022). A Novel Glutamine Metabolism-Related Gene Signature in Prognostic Prediction of Osteosarcoma. Int. J. Gen. Med. 15, 997–1011. 10.2147/ijgm.s352859 35136353 PMC8817953

[B45] Wang W. Zou W. (2020). Amino Acids and Their Transporters in T Cell Immunity and Cancer Therapy. Mol. Cell 80 (3), 384–395. 10.1016/j.molcel.2020.09.006 32997964 PMC7655528

[B46] Wong C. C. Qian Y. Li X. Xu J. Kang W. Tong J. H. (2016). SLC25A22 Promotes Proliferation and Survival of Colorectal Cancer Cells with KRAS Mutations and Xenograft Tumor Progression in Mice via Intracellular Synthesis of Aspartate. Gastroenterology 151 (5), 945–960. 10.1053/j.gastro.2016.07.011 27451147

[B47] Yan G. An Y. Xu B. Wang N. Sun X. Sun M. (2021). Potential Impact of ALKBH5 and YTHDF1 on Tumor Immunity in Colon Adenocarcinoma. Front. Oncol. 11, 670490. 10.3389/fonc.2021.670490 34079761 PMC8165310

[B48] Yuan H. Liu J. Zhao L. Wu P. Chen G. Chen Q. (2021). Prognostic Risk Model and Tumor Immune Environment Modulation of m5C-Related LncRNAs in Pancreatic Ductal Adenocarcinoma. Front. Immunol. 12, 800268. 10.3389/fimmu.2021.800268 34956238 PMC8692582

[B49] Zhao Y. Zhang J. Wang S. Jiang Q. Xu K. (2021). Identification and Validation of a Nine-Gene Amino Acid Metabolism-Related Risk Signature in HCC. Front. Cell Dev. Biol. 9, 731790. 10.3389/fcell.2021.731790 34557495 PMC8452960

[B50] Zhou W. Feng X. Ren C. Jiang X. Liu W. Huang W. (2013). Over-expression of BCAT1, a C-Myc Target Gene, Induces Cell Proliferation, Migration and Invasion in Nasopharyngeal Carcinoma. Mol. Cancer 12, 53. 10.1186/1476-4598-12-53 23758864 PMC3698204

