# Supplementary figures and images for: Analysis of the Prognostic Significance and Immune Infiltration of the Amino Acid Metabolism-Related Genes in Colon Adenocarcinoma
Source: Front Genet. 2022 Aug 10;13:951461. doi: 10.3389/fgene.2022.951461 (PMC9399353; doi:10.3389/fgene.2022.951461)

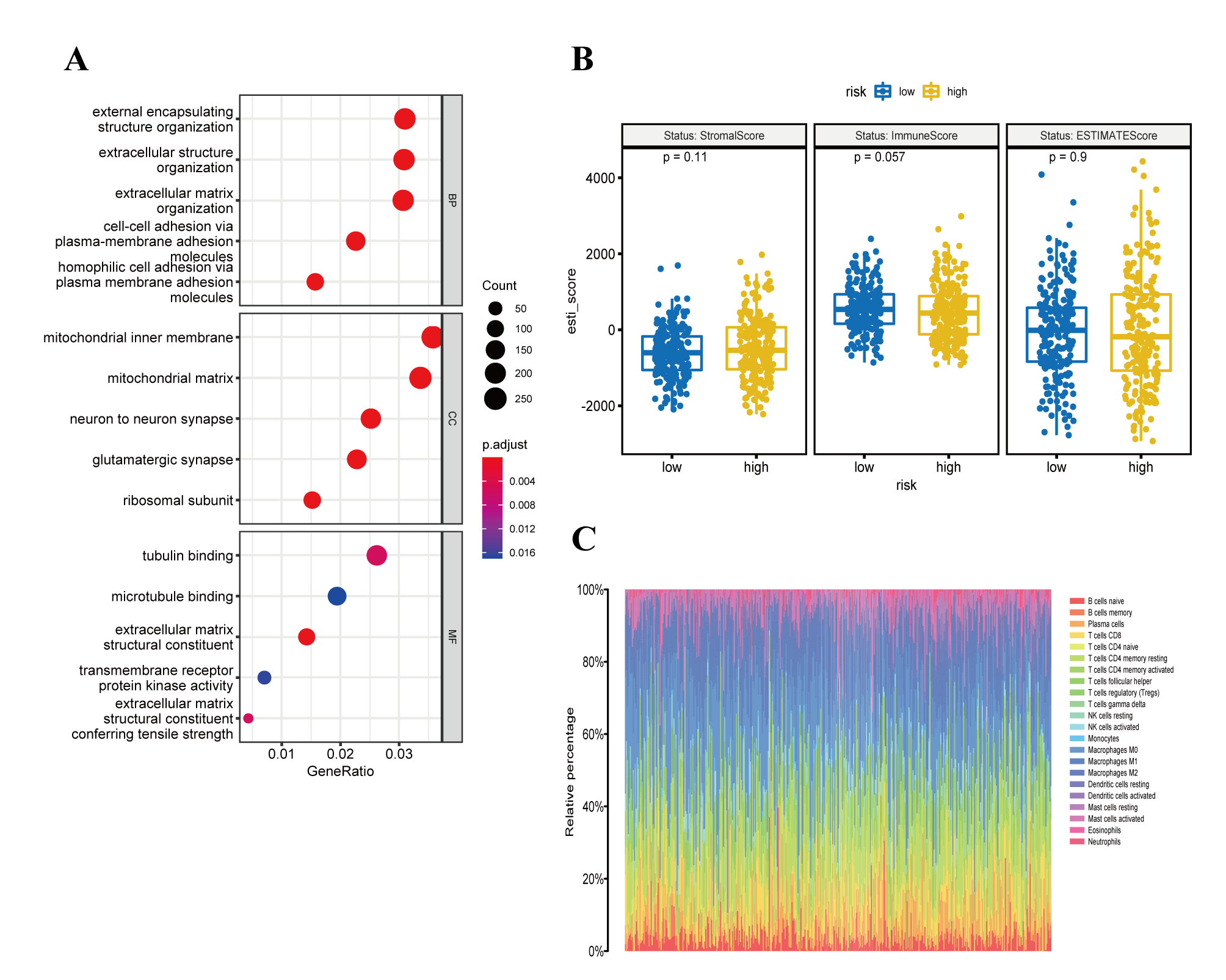

Supplement: Supplementary file 4 [file Image3.TIF]

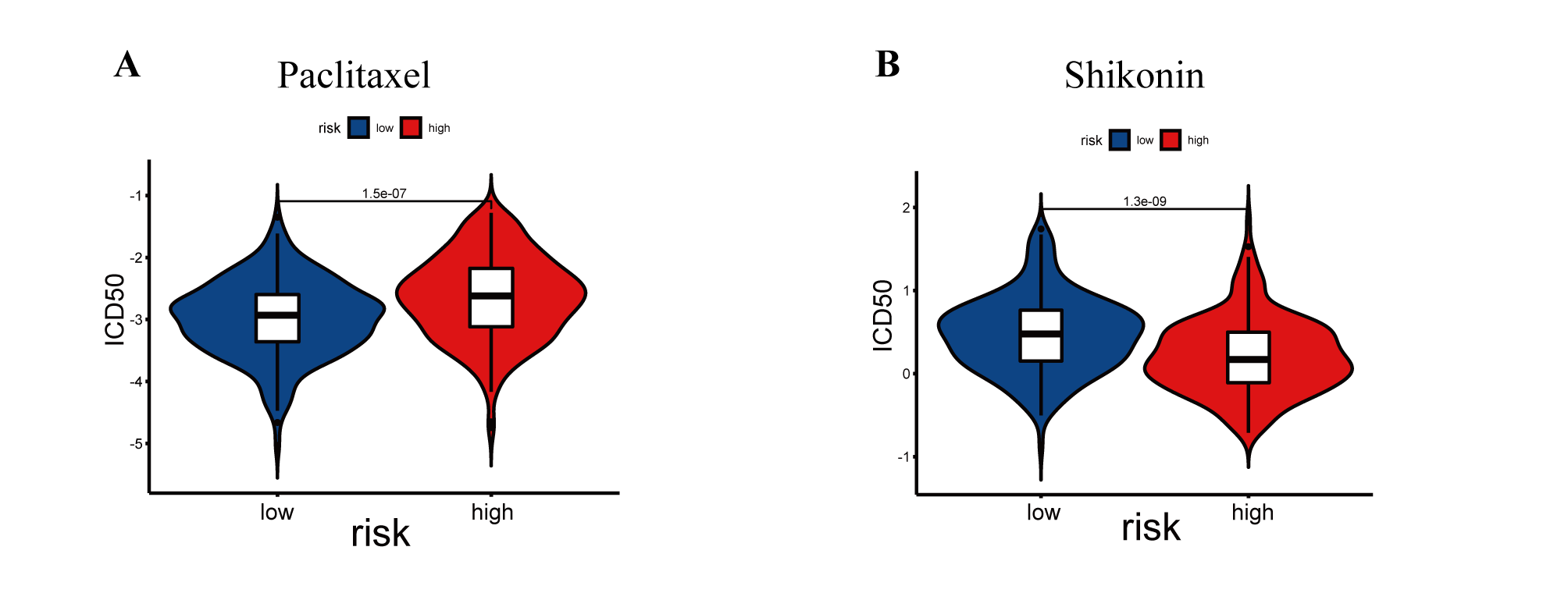

Supplement: Supplementary file 5 [file Image4.TIF]

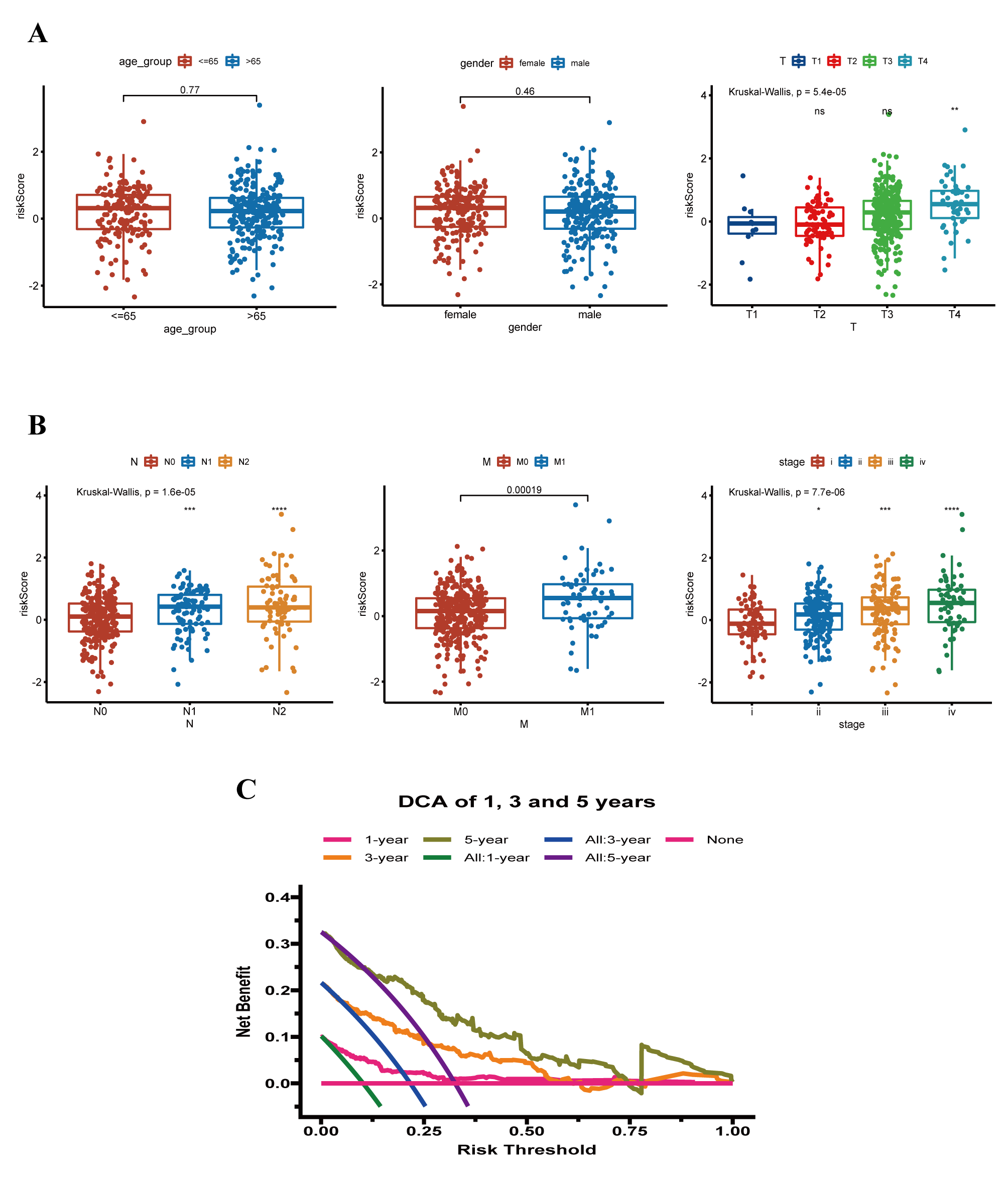

Supplement: Supplementary file 7 [file Image2.TIF]

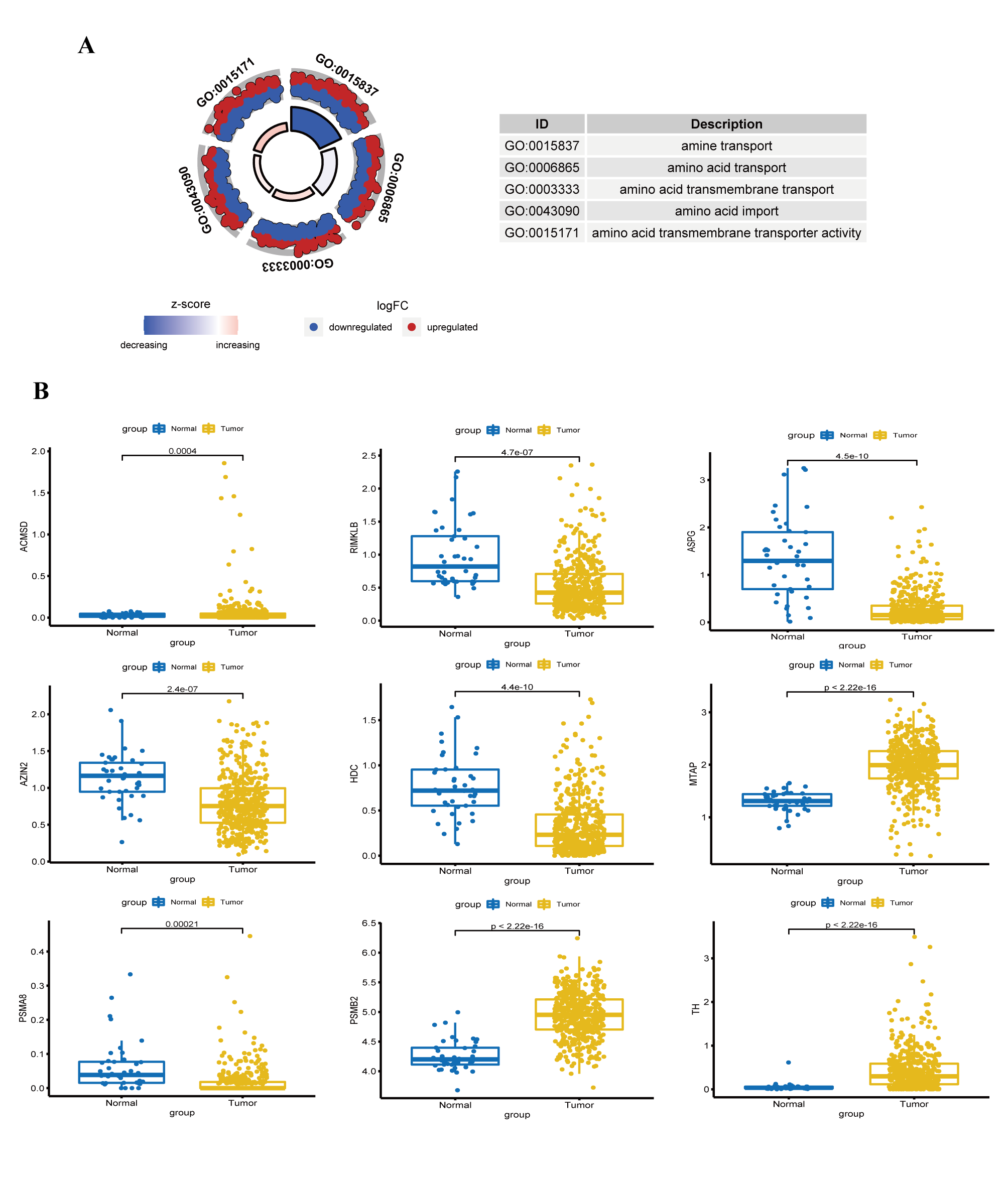

Supplement: Supplementary file 9 [file Image1.TIF]
